# The Roles of TRIMs in Antiviral Innate Immune Signaling

**DOI:** 10.3389/fcimb.2021.628275

**Published:** 2021-03-15

**Authors:** Zhou Shen, Lin Wei, Zhi-bo Yu, Zhi-yan Yao, Jing Cheng, Yu-tong Wang, Xiao-tian Song, Miao Li

**Affiliations:** ^1^Key Laboratory of Immune Mechanism and Intervention on Serious Disease in Hebei Province, Department of Immunology, Hebei Medical University, Shijiazhuang, China; ^2^Center Laboratory, Affiliated Hospital of Hebei University, Baoding, China

**Keywords:** E3 ubiquitin ligase, tripartite motif (TRIM), innate immune response, signaling pathway, direct game

## Abstract

The Tripartite motif (TRIM) protein family, which contains over 80 members in human sapiens, is the largest subfamily of the RING-type E3 ubiquitin ligase family. It is implicated in regulating various cellular functions, including cell cycle process, autophagy, and immune response. The dysfunction of TRIMs may lead to numerous diseases, such as systemic lupus erythematosus (SLE). Lots of studies in recent years have demonstrated that many TRIM proteins exert antiviral roles. TRIM proteins could affect viral replication by regulating the signaling pathways of antiviral innate immune responses. Besides, TRIM proteins can directly target viral components, which can lead to the degradation or functional inhibition of viral protein through degradative or non-degradative mechanisms and consequently interrupt the viral lifecycle. However, new evidence suggests that some viruses may manipulate TRIM proteins for their replication. Here, we summarize the latest discoveries on the interactions between TRIM protein and virus, especially TRIM proteins’ role in the signaling pathway of antiviral innate immune response and the direct “game” between them.

## Introduction

**Figure 1:**
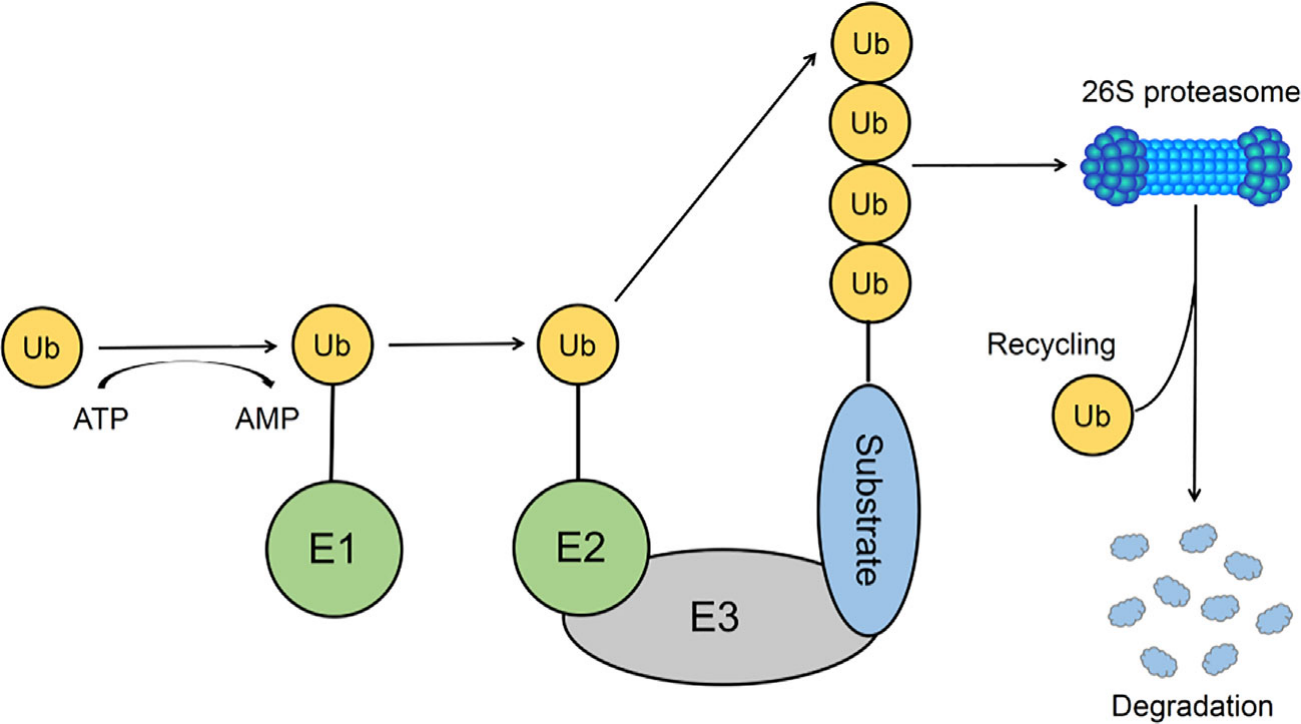
The Ubiquitin-Proteasome System. The conjugation reaction of ubiquitin is catalyzed by the E1 ubiquitin-activating enzyme, E2 ubiquitin-conjugating enzymes, and E3 ubiquitin ligases. E3 ubiquitin ligase could recognize substrates and transfer ubiquitin from E2 ubiquitin-conjugating enzymes to substrates, resulting in the proteasome degradation of the polyubiquitinated substrates.

Ubiquitination is an extraordinary essential post-transcriptional modification process that functions in both innate and adaptive immune responses. The key enzymes required for the ubiquitination process are classified into ubiquitin-activating enzyme E1, ubiquitin-conjugating enzymes E2, and ubiquitin ligases E3 (Pickart and Eddins, 2004; Chernorudskiy and Gainullin, 2013) (**Figure 1**). There are many E3 ligases in human cells due to their capability to recognize specific substrates and transfer ubiquitin from E2 enzymes to those substrates (Davis and Gack, 2015). The E3 ubiquitin ligase superfamily can be classified into three categories according to their specific domains: really interesting new gene (RING) family, homologous to E6-AP C-terminus associated protein (HECT) family (Berndsen and Wolberger, 2014; Mattiroli and Sixma, 2014), and those of unclassified type.

**Figure 2:**
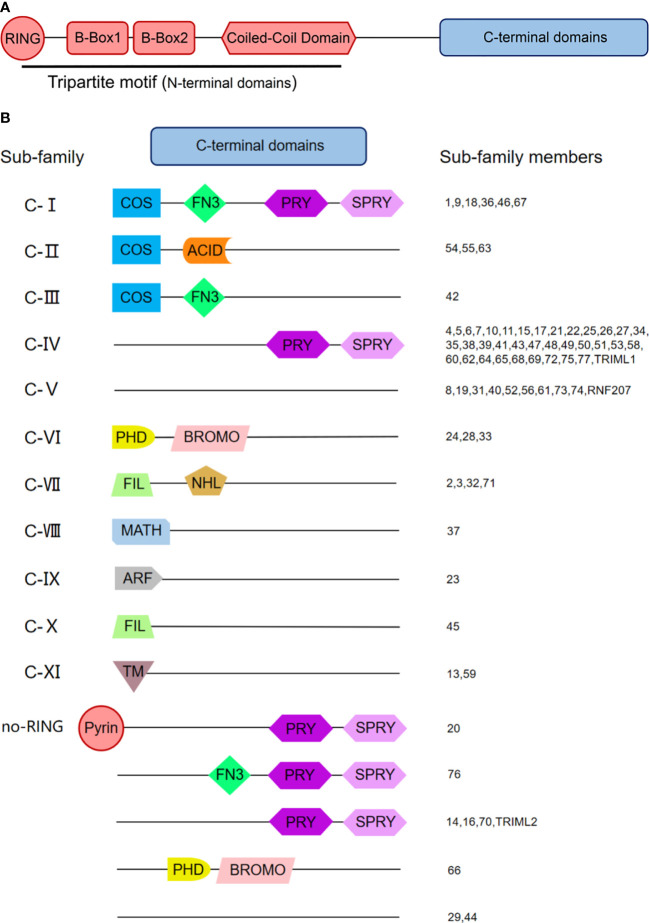
The Domain Structure and the Classification of Tripartite Motif (TRIM) Family Proteins. **(A)** Domain structure of TRIM proteins. Most TRIM proteins possess conservative RBCC domains at the amino-terminus and diverse domains at the carboxyl-terminus. RBCC domains consist of a RING finger domain, a B-box 1 and/or B-box 2 domain, and a coiled-coil domain (CCD). **(B)** Classification of TRIM proteins. TRIM proteins with the RING domain are classified into 11 subfamilies from Class I to Class XI according to their distinctive C-terminal domains. Besides, there is an unclassified group lacking the RING domain (no RING). PRY, SPRY-associated domain; SPRY, SPIa and the ryanodine receptor domain; COS, C-terminal subgroup one signature domain; FN3, fibronectin type 3 domain; ACID, acid-rich region; PHD, plant homeodomain; BROMO, bromodomain; FIL, filamin-type IG domain; NHL, NHL repeats; MATH, meprin, and tumor necrosis factor receptor-associated factor (TRAF) homology domain; ARF, ADP-ribosylation factor family domain; TM, transmembrane region. Numbers indicate individual TRIM proteins.

The tripartite motif (TRIM) proteins are a highly conserved superfamily of proteins, which is the largest subfamily of the RING-type E3 ubiquitin ligase family (Esposito et al., 2017). TRIM protein family is named for its specific RBCC domains at the amino-terminus, consisting of a RING finger domain at the amino-terminus, one or two B-Box domains, and a coiled-coil domain (CCD) (**Figure 2A**). The RING domain is essential for TRIM to exert its E3 ubiquitinase catalytic activity. This domain contains two “zinc finger” structures that recognize and transfer the ubiquitins from ubiquitin-binding enzyme E2 to other proteins. However, not all TRIMs proteins contain the RING finger domain, such as TRIM14, TRIM29, TRIM44, etc. The second domain at the amino-terminus of TRIM protein is the B-Box domain. Although the B-Box domain contains the “zinc finger” structure, it generally doesn’t exert E3 ubiquitin ligase activity. There are two different subtypes of B-Box: B-Box1 and B-Box2. Most TRIM proteins contain one B-Box2 domain or those two B-Box domains, while a few TRIMs, such as TRIM69, don’t have any of them. So far, the function of the B-Box domain is still a mystery. The B-Box domain is assumed to be involved in the TRIM’s assembly and interactions between TRIM proteins and others.

In some cases, this domain can even exert the E3 ubiquitin ligase activity [such as TRIM18 (Massiah et al., 2006)]. The following CCD domain is a superhelix structure that is generally considered to mediate homo and heterodimerization of TRIM proteins, which is essential for biological function (Reymond et al., 2001; Li et al., 2011b). CCD domain forms an antiparallel dimer that can increase the molecular stability of TRIM proteins, seeming like a common feature of TRIMs (Li et al., 2014; Weinert et al., 2015).

Besides the domains at amino-terminus, those at carboxyl-terminus are also diverse, including SPRY-associated domain (PRY), SPIa and the ryanodine receptor domain (SPRY), C-terminal subgroup one signature domain (COS), fibronectin type III repeats domain (FNIII), etc. According to the various domains in the carboxyl-terminal region, TRIM protein family members are divided into 11 subgroups from Class I to Class XI comprising about 80 members. Besides, there is an unclassified group lacking the RING domain (no RING) (**Figure 2B**). Recent studies have shown that these domains may play unique roles. For example, the PRY-SPRY domain of TRIM21 (also known as B30.2 domain) can bind to the Fc segment of immunoglobulin (Ig) and limit the spread of the non-enveloped virus such as adenovirus (Marina et al., 2013; Rhodes and Isenberg, 2017). TRIM23, as the only protein possessing ADP-ribosylation factor (ARF) family domain among family members, have both E3 ubiquitin ligase activity and GTPase activity. TRIM23 can activate TANK-binding kinase 1 (TBK1) through its GTPase activity and then induce autophagy and target viral proteins for degradation (Kmj et al., 2017).

TRIM proteins could play multiple regulatory roles in various cellular processes, particularly in innate immune responses and carcinogenesis. The RING domain of TRIM proteins confers them E3 ligase activity, which can mediate ubiquitination, ISGylation, or SUMOylation of specific substrates (Chu and Yang, 2011; Khanna et al., 2018; Wu et al., 2020). These post-translational modifications can lead to the degradation of the substrates *via* the lysosomal or proteasomal pathways (Khanna et al., 2018; Wu et al., 2019a). Recent studies have shown that RING-type E3 ubiquitin ligase can also ubiquitinate some signaling proteins in a “non-degradative” way, thereby regulating their activity or subcellular localization (Didier et al., 2003; Liu et al., 2017). In this review, we’d like to highlight the interactions between TRIM protein and virus. We mainly focus on the following two aspects: TRIM proteins’ roles in regulating the signaling pathway of innate immune responses and the direct “game” between virus and TRIM proteins.

## The Roles of TRIM Proteins in the Signaling Pathway of Innate Immune Responses

**Figure 3:**
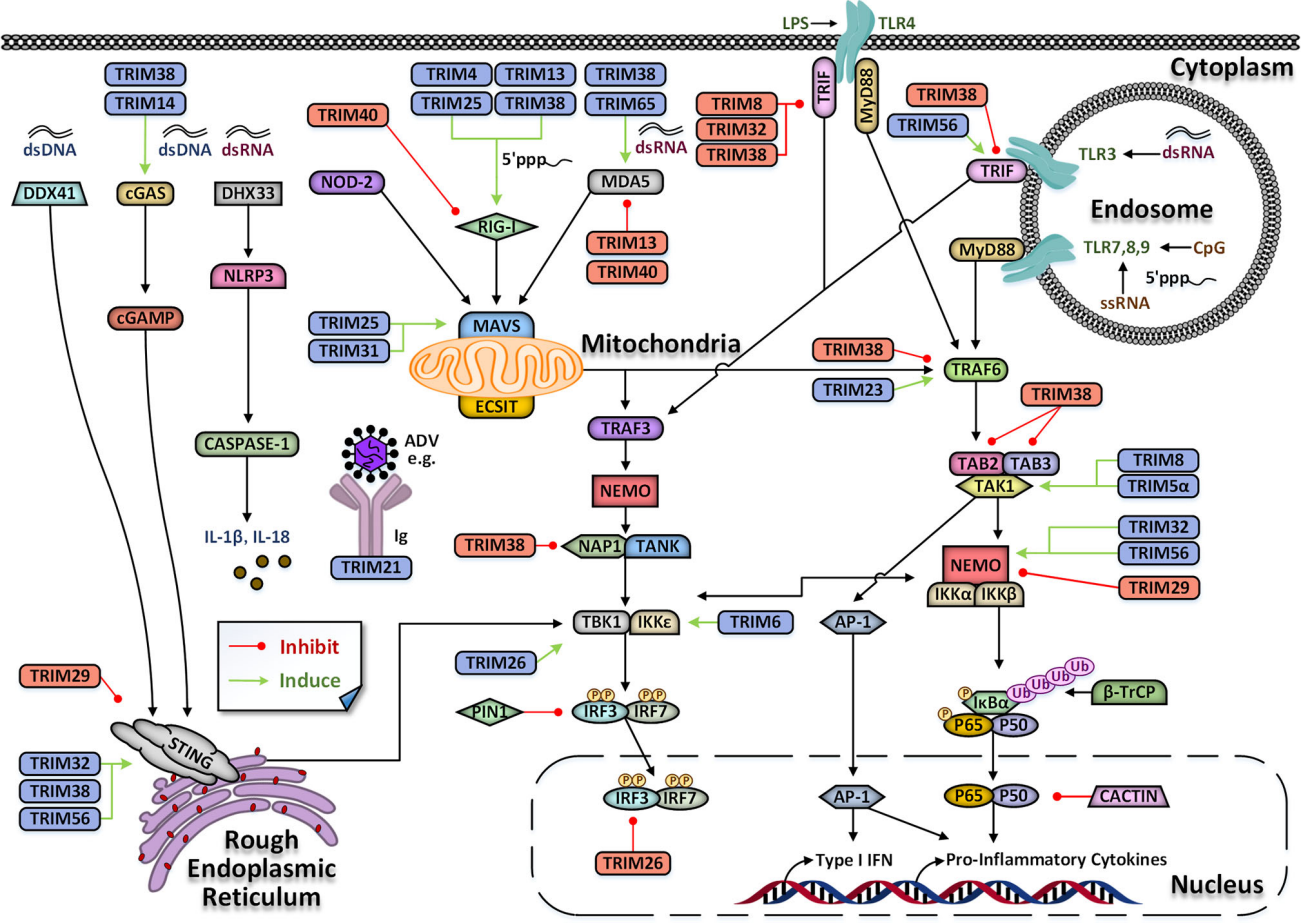
TRIM-mediated regulation of innate immune signaling pathways. TRIM proteins play a dual role in antiviral immune signaling pathways indicated by black arrows. They could positively or negatively regulate antiviral immune signaling pathways, indicated by green arrows or red lines, respectively. Also, some TRAM proteins can act as common pathogen PRRs, or as cytosolic Fc receptors (such as TRIM21) to recognize non-enveloped viruses bound by immunoglobulin (Ig). DDX41, DEAD-box helicase 41; cGAS, cyclic GMP-AMP synthase; DHX33, DEAH-box helicase 33; NOD-2, nucleotide-binding oligomerization domain-containing protein 2; RIG-I, retinoic acid-inducible gene I; MDA5, melanoma differentiation-associated protein; TLR, Toll-like receptors; STING, stimulator of IFN genes; MAVS, mitochondrial antiviral signaling protein; TAK1, TGF-β-activated kinase 1; TAB2, TAK1/MAP3K7-binding protein 2; MyD88, Myeloid differentiation primary response gene 88; TRIF, TIR-domain-containing adapterinducing interferon-β; NEMO, NF-κB essential modulator; NAP-1, nucleosome assembly protein; TNF, tumor necrosis factor; TRAF, TNF receptor-associated factors; TANK, TANK-binding kinase 1; IκB, inhibitor of NF-κB; IKK, IκB kinase; TBK1, TANK binding kinase 1; IFN, interferon; IRF, interferon regulatory factor; P, phosphorylation; Ub, ubiquitin; dsDNA, double-stranded DNA; dsRNA, double-stranded RNA; ssRNA, single-stranded; ADV, adenovirus; IL, interleukin; AP-1, activator protein-1; PIN1, Peptidyl-prolyl cis-trans isomerase NIMA-interacting 1; ECSIT, evolutionarily conserved signaling intermediate in Toll pathway; CASPASE-1, cysteinyl aspartate specific proteinase 1; β-TrCP, β-transducinrepeats containing proteins.

The innate immunity makes up the first line of defense against the invasion of pathogens. Upon invasion by pathogens, pathogen-associated molecular patterns (PAMPs) are sensed by pattern recognition receptors (PRRs) of innate immune cells, including retinoic acid-induced gene-I-like receptor (RLRs), Toll-like receptors (TLRs), and cytosolic DNA receptors (Brennan and Bowie, 2010; Cai et al., 2014; Brubaker et al., 2015). Triggering of PRRs culminates in the activation of various signaling pathways and the transcriptional induction of proinflammatory cytokines and type I interferons (IFNs), which together coordinate antimicrobial immune defenses (Takeuchi and Akira, 2010; Paludan et al., 2011). Protein post-translational modifications (PTMs), including phosphorylation, methylation, ubiquitination, and acetylation, regulate this process (Liu et al., 2018; McFadden and Horner, 2020; Song et al., 2020; Wu and Li, 2020; Zhong et al., 2020). TRIM protein family has E3 ubiquitin ligase activity, providing a structural basis for participating in post-translational modification of proteins and regulating innate immune response, especially antiviral innate immune response (**Figure 3**).

At present, many studies have shown that there are mainly three innate immune signal pathways regulated by TRIM protein, namely, TLR signaling pathway, RLR signaling pathway, and cyclic GMP-AMP synthase (cGAS)- stimulator of IFN genes (STING) pathway.

### The Roles of TRIM Protein in the TLR Signaling Pathways

The sensors of TLR signaling pathways are TLRs that exist on the surface of the cell membrane or in the intracellular compartment. They sense nucleic acid, lipid, or protein components derived from pathogens. TLR family members are generally divided into six subfamilies based on their primary structure, which are TLR1 subfamily (including TLR1 and TLR2), TLR3 subfamily, TLR4 subfamily, TLR5 subfamily, TLR7 subfamily (including TLR7, TLR8, and TLR9), and TLR11 subfamily (including TLR11, TLR12, and TLR13). TLR3, TLR7, TLR8, and TLR9 are mainly involved in viral recognition (Gent et al., 2018). Upon binding to the ligand, TLRs dimerize and trigger the activation of signaling cascade reaction, ultimately resulting in the production of proinflammatory cytokines and antiviral type I interferons (IFNs) mediated by nuclear factor kappa-B (NF-κB) and interferon regulatory factor (IRF). TLRs recruit kinase IL-1 receptor-associated kinase 1/4 (IRAK1/4) and E3 ubiquitin ligase TNF receptor-associated factor 6 (TRAF6) *via* adaptor molecule Myeloid differentiation primary response gene 88 (MyD88). TRAF6 catalyzes auto-ubiquitination. Ubiquitinated TRAF6 recognizes TAK1/MAP3K7-binding protein 2 (TAB2) and activates TGF-β-activated kinase 1 (TAK1), and ultimately results in activation of IκB kinase α/β/γ (IKKα/β/γ) and NF-κB (Kishida et al., 2005). TLR3 is such a unique receptor that can recognize double-stranded RNA and poly(I:C). TLR3 transmits signals *via* adaptor molecule TRIF (TIR-domain-containing adaptor inducing IFNβ), but not MyD88, which leads to activation of transcription factors interferon regulatory factor 3 (IRF3) and interferon regulatory factor 7 (IRF7) through TNF receptor-associated factor 3 (TRAF3) and transforming growth factor-β- activated kinase 1/IκB kinase ϵ (TBK1/IKKϵ) (Yamamoto et al., 2002; Yamamoto et al., 2003). A lot of TRIM proteins can regulate the response process by targeting these signaling molecules. For instance, TRIM8 inhibits TRIF-mediated signaling transduction by disrupting TRIF-TBK1 interaction (Ye et al., 2017). TRIM32 could negatively regulate TLR3/4-mediated immune responses by targeting TRIF to TAX1 binding protein 1 (TAX1BP1)-mediated selective autophagic degradation (Yang et al., 2017). TRIM38 could regulate the stability of signaling transduction proteins including TRIF (Qinghua et al., 2012), TRAF6 (Zhao et al., 2012), nucleosome assembly protein 1(NAP1) (Zhao et al., 2012), TAB2/3 (Ming-Ming et al., 2014), and then negatively regulate TLR signaling. TRIM29 induces NF-κB essential modulator (NEMO) degradation and negatively regulates the production of proinflammatory cytokines in alveolar macrophages (Xing et al., 2016). Besides above TRIM proteins that negatively regulate the TLR/TRIF pathway, TRIM56 were found to positively regulate the TLR3-mediated interferon pathway through interacting with TRIF, which acts in an E3-ligase-independently mechanism (Shen et al., 2012). Similar to TLR signal pathways, TNFα and IL-1β also transmit signals *via* the MyD88 molecule. TRIM8 can enhance TNFα and IL-1β signaling transduction through mediating K63-linked polyubiquitin modification of TAK1 (Li et al., 2011a).

### The Role of TRIM Protein in the RLR Signaling Pathway

RLR consists of three members: retinoic acid-inducible gene I (RIG-I), melanoma differentiation-associated protein 5 (MDA5), and laboratory of genetics and physiology 2 (LGP2). RLRs harbor two N-terminal caspase recruitment domains (CARDs) excepting LGP2, a central DExD/H-box helicase domain and a C-terminal regulatory domain (RD). With the unique DExD/H-box RNA helicase activity, they could sense single- or double-stranded RNA from the virus to initiate immune response (Goubau et al., 2013). When RIG-I and MDA5 recognize and bind PAMPs, they undergo a conformational change that exposes their caspase recruitment domain (Bharaj et al.) to interact with mitochondrial antiviral signal transduction protein (MAVS). They could then trigger the downstream NF-κB signaling pathway and the IFN signaling pathway (Seth et al., 2005; Hou et al., 2011). Although LGP2 lacks the CARD domain for signal transduction, it could act as a positive regulator of RIG-I and MDA5-mediated antiviral responses by facilitating viral RNA recognition by RIG-I and MDA5 with its ATPase domain (Satoh et al., 2010; Bruns et al., 2014).

RIG-I and MDA5 are strictly regulated by post-translational modification, which ensures the rapid activation of RLR and prevents excessive immune response (Chiang and Gack, 2017). TRIM25 is the first identified immunomodulatory factor of the TRIM protein family. The SPIa and the ryanodine receptor (SPRY) domain at the carboxyl-terminus of TRIM25 (Gack et al., 2007) catalyzes the K63-linked polyubiquitination of the CARDs domain of RIG-I, induces RIG-I oligomerization to integrate with MAVS, and then triggers downstream signaling transduction (Zeng et al., 2010). The stability of TRIM25 protein is precisely regulated by K48-linked ubiquitination, which induces degradation of TRIM25 *via* the proteasome (Inn et al., 2011). Ubiquitin specific peptidase 15 (USP15) could enhance the stabilization of TRIM25 by counteracting its K48-linked ubiquitylation and thereby positively regulate the TRIM25-RIG-I signaling pathway (Eva-Katharina et al., 2011). However, the role of USP15 in those responses remains controversial, and it has been demonstrated to act as an inhibitor of the RIG-I mediated interferon signaling pathway through deconjugating the Lys63-linked polyubiquitin chains from RIG-I or physically sequestering the RIG-I-MAVS interaction (Zhang et al., 2015). TRIM25 can also be targeted by nonstructural protein 1 (NS1) of influenza A virus (IAV), thus losing TRIM25’s positive regulatory abilities in RIG-I ubiquitination and RIG-I signal transduction (Gack et al., 2009). The ability of NS1 protein to inhibit RIG-I signaling differs markedly between influenza A virus subtypes. However, no matter whether NS1 can block RIG-I activation, it can bind to TRIM25 protein (Kuo et al., 2010). Furthermore, another study confirmed that influenza A Virus NS1 protein interacts with TRIM25 in a species-specific manner. Although NS1 protein cannot bind mouse TRIM25, it confers its ability to block IFN production in mice (Rajsbaum et al., 2012). Those results demonstrate that NS1-TRIM25 regulation does not necessarily lead to the differential induction of IFNs by influenza viruses and indicate that other host proteins (except TRIM25) may be involved in regulating RIG-I signaling and be targeted by NS1 of influenza A virus. Additionally, several studies have uncovered some host proteins, such as Nuclear Dbf2-related kinase 2 (NDR2), Nucleotide-binding oligomerization domain, Leucine-rich repeat and pyrin domain containing 12 (NLRP12), Caspase-12 and Lnczc3h7a, could regulate the E3 ligase activity of TRIM25 (Wang et al., 2010; Chen et al., 2019; Lin et al., 2019; Liu et al., 2019b).

However, the positive regulatory role of TRIM25 in RIG-I signaling is only observed when they use exogenous TRIM25. Some studies showed that removing endogenous TRIM25 has no impact on RIG-I activity in response to either RNA ligands or infection with influenza virus or SeV (Shi et al., 2017; Cadena et al., 2019; Hayman et al., 2019). In contrast, the removal of endogenous Riplet has a profound effect on RIG-I signaling, indicating that Riplet, but not TRIM25, is essential for RIG-I ubiquitination and its signaling transduction (Hayman et al., 2019). The current dogma stating a major requirement for Trim25 as a positive regulator of RIG-I signaling may need to be revised.

Other TRIMs proteins, such as TRIM4, TRIM21, TRIM26, TRIM40, and TRIM65, also play crucial roles in regulating RLRs-mediated innate immune response. TRIM4 is a positive regulator of RIG-I mediated IFN induction by targeting RIG-I for K63-linked polyubiquitination (Yan et al., 2014). TRIM21 could interact with MAVS and catalyzes the K27-linked polyubiquitination of MAVS, thereby promoting the recruitment of TBK1 to MAVS and positively regulate innate immune response (Xue et al., 2018). TRIM26 has also been reported to regulate MAVS-mediated IRF3 activation during RNA virus infection. RNA virus-triggered TRIM26 autoubiquitination could bridge TBK1-NEMO interaction, which is essential for recruiting TBK1 to the MAVS signalosome and its activation. Activated TBK1 dissociates from TRIM26, phosphorylates IRF3, and induces type I IFNs (Ran et al., 2016). However, there are also debates regarding the role of TRIM26 in IFN-β production and antiviral immune response. Gao’s group demonstrated that TRIM26 negatively regulated the production of IFN-β by targeting nuclear IRF3 and promoting its K48-linked polyubiquitination and degradation in the nucleus (Wang et al., 2015). This dual effect of TRIM26 may be related to its cellular localization. Cytoplasmic and nuclear TRIM26 could target different proteins and ubiquitinate them with different forms to exert their different roles. TRIM40 could negatively regulate both MDA5- and RIG-I-induced signal pathways by promoting K27- and K48-linked ubiquitination of MDA5 and RIG-I, respectively, and increasing their proteasomal degradation (Zhao et al., 2017). TRIM65 is essential for MDA5-induced IRF3 activation by specifically interacting with MDA5 and promoting K63-linked ubiquitination of MDA5, critical for MDA5 oligomerization and activation (Lang et al., 2017; Meng et al., 2017).

Interestingly, TRIM13 exerts an opposite regulatory effect on RIG-I (Versteeg et al., 2013) and MDA5 (Narayan et al., 2014) signaling pathway. Although the precise molecular mechanism hasn’t been clarified, the possible reasons are as follows: Firstly, The dual-directional regulation of TRIM13 on RIGI and MDA5 may be related to the time course of infection. A recent study has shown that MDA5 can substantially activate interferon regulatory factor 3 during infection by Paramyxoviruses. TRIM13 could initiate the innate immune response in the early stage of infection by positively regulating the RIG-I pathway. TRIM13 may inhibit the effect of persistent activation of MDA5 on RIG I by negatively regulating the MDA5 pathway, which represses excessive immune response and avoids immunopathological damage in the late stage of infection. Secondly, The targets of TRIM13 regulating the RIG-I pathway and MDA5 pathway may be different. SUMOylation could stabilize the substrate by inhibiting the K48-linked polyubiquitination modification. Although TRIM38 acts as a ubiquitin ligase, a recent report have shown that it also has E3 SUMO ligase activity and positively regulates antiviral immune response by sumoylating RIG-I and MDA5 and suppressing their K48-linked polyubiquitination and degradation in early-infected cells (Hu et al., 2017a).

TRIM14, being one of the few TRIM protein family members that lack the RING domain, performs regulatory function independent of E3 ubiquitin ligase activity. It can provide a docking platform for the assembly of the mitochondrial signaling complex Werner helicase protein (WHIP)-TRIM14-protein phosphatase PPP6C, which is essential for RIG-I K63 ubiquitination and activation of RIG-I-mediated innate antiviral immunity (Zhou et al., 2014; Tan et al., 2017). During RNA virus infection, TRIM31 can mediate K63-linked polyubiquitination on MAVS at multiple sites, promoting MAVS to form aggregates and inducing type I interferon production (Liu et al., 2016b).

### The Role of TRIM Protein in the cGAS-STING Signaling Pathway

Studies have confirmed that the cGAS-STING signaling pathway mainly recognizes DNA viruses. The cGAS possessing anti-retroviral activity is essential for the recognition of DNA viruses. Upon recognizing viral DNA, cGAS produces the second messenger cyclic guanosine monophosphate-adenosine monophosphate (cGAMP), which subsequently activates STING and induce type I interferon production (Chen et al., 2016b). TRIM38 can induce SUMOylation of cGAS and STING (Hu et al., 2016), thereby preventing K48-linked polyubiquitination and proteasome-mediated degradation of cGAS. Herpes simplex virus (HSV-1) induces TRIM14 expression and recruits the deubiquitinating enzyme USP14, which inhibits the K48-linked polyubiquitination of cGAS to increase its stability and enhances the immune response against HSV-1 (Chen et al., 2016a). TRIM29, which is specifically expressed in alveolar macrophages and airway epithelial cells, negatively regulates the immune response against DNA viruses by mediating polyubiquitination and STING degradation (Xing et al., 2017). It has been previously reported that TRIM56 and TRIM32 can promote K63-linked polyubiquitination of STING and increase antiviral response during DNA virus infection (Tsuchida et al., 2010; Zhang et al., 2012). However, recent studies have shown that TRIM56 and TRIM32 do not directly ubiquitinate STING. They can synthesize ubiquitin chains that bind to NEMO and presumably mediate NEMO’s ubiquitination to activate IKKβ, finally leading to NF-κB and TBK1 IRF3 activation (Qiang et al., 2014; Fang et al., 2017; Motwani et al., 2019).

Besides, many TRIM proteins directly regulate the downstream interferon signaling pathway, which can work in coordination with the three signaling pathways mentioned above to precisely regulate the antiviral immune response.

## TRIM Proteins Play a Direct “Game” With Viruses

### TRIM Proteins Directly Antagonize the Virus by Targeting Viral Components

Upon viral invasion, the sensors in the cell membrane and the cytoplasm could recognize the virus to initiate a series of response mechanisms. TRIM proteins play a regulatory role in the innate viral immune response pathway and directly antagonize the virus. TRIM5α, which can inhibit the infection of retroviruses such as Human Immunodeficiency Virus 1 (HIV-1) and N-tropic murine leukemia virus (N-MLV), maybe the first and the most widely-studied protein in these aspects (Perron et al., 2004; Stremlau et al., 2004; Yap et al., 2004). According to the current research results, TRIM5α may form a complementary lattice with the viral nucleocapsid hexamer lattice, which induces the premature disassembly of viral capsid disintegration of immature virus particles possibly through ubiquitin degradation, and then inhibits viral replications (Stremlau et al., 2004; David et al., 2005; Campbell et al., 2015). TRIM5α also acts as a selective autophagy receptor mediating HIV-1 restriction. On the one hand, TRIM5α promotes autophagy by acting as a platform for the assembly of active ULK1 and BECN1 complexes. On the other hand, TRIM5α acts as a selective autophagy receptor targeting HIV-1 p24 to autophagosome and promoting its degradation (Mandell et al., 2014a; Mandell et al., 2014b). Besides, viral PAMPs are released to activate the innate immune signaling pathway (Kutluay et al., 2013). A recent study shows that TRIM5α can restrict flavivirus replication by targeting the viral protease for proteasomal degradation (Chiramel et al., 2019). TRIM5α has also been reported to act as a receptor to bind viral nucleocapsid, catalyze the formation of unanchored K63 polyubiquitin chains, and finally activate the signaling pathways mediated by TAK1, activator protein 1 (AP1), and NF-κB (Thomas et al., 2011). And this kind of restriction effects of TRIM5α on retroviruses appears to be relatively obvious in primates (Matthew et al., 2004), indicating that TRIM5 plays its role in specific species.

Besides TRIM5α, a multitude of other TRIM proteins have been shown to antagonize the virus directly. TRIM56 could act as an antiviral host restriction factor to inhibit the replication of members of the *Flaviviridae family*, including Bovine viral diarrhea virus (BVDV), dengue virus serotype 2 (DENV2), and yellow fever virus (YFV) (Wang et al., 2011; Liu et al., 2014). Beyond members of the *Flaviviridae family*, TRIM56 also inhibits human corona virus (HCoV) OC43 replication (Liu et al., 2014). Furthermore, TRIM56’s antiflavivirus effects required both the E3 ligase activity that lies in the N-terminal RING domain and the integrity of its C-terminal portion, while the restriction of HCoV-OC43 relied upon the TRIM56 E3 ligase activity alone. Distinct TRIM56 domains may confer differing antiviral mechanisms. Current researches has demonstrated that TRIM56 inhibits YFV/DENV2/BVDV replication by impairing intracellular viral RNA replication, whereas it curbs HCoV-OC43 progeny viral yield by targeting viral packaging and release stages but not intracellular viral RNA accumulation (Wang et al., 2011; Liu et al., 2014). C-terminal region of TRIM proteins mediate protein-protein or protein-RNA interactions between TRIMs and cellular and/or viral proteins/RNA, and may hinder viral RAN replicaiton. Besides, TRIM proteins’ E3 ligase activity may modulate a posttranslational modification of viral proteins and/or host factors to suppress positive-strand RNA virus replication. We speculated that TRIM56 may antagonize different viruses by targeting different viral or host protein. However, the interaction partners of TRIM56 remain to be elucidated. Consistent with previous researches, recent studies have shown that carboxy-terminal domains of TRIM56 inhibit IAV and Influenza B virus (IBV) replication by blocking viral RNA synthesis (Liu et al., 2016a). Similarly, TRIM56 could also be a host restriction factor of the Zika virus, depending on its RNA-binding activity (Yang et al., 2019). TRIM22 can restrict retrovirus by interfering with viral Gag protein transport by regulating transcription factor specificity protein 1 (Sp1) (Sp1) (Kajaste-Rudnitski et al., 2011; Turrini et al., 2015). Besides, TRIM22 can inhibit IAV and encephalomyocarditis virus (EMCV) by targeting viral nuclear proteins and 3C proteases, respectively (Eldin et al., 2009; Di Pietro et al., 2013). TRIM19, also known as promyelocytic leukemia (PML) protein, is a key component of the PML nucleosome (Ishov et al., 1999). TRIM19 can restrict many DNA virus replication, including herpes virus and adenovirus and RNA viruses such as IAV and vesicular stomatitis virus (VSV). The mechanism by which TRIM19 inhibits the virus is multiple. TRIM19 can epigenetically silence the viral genome and entrap the newly synthesized viral nucleocapsid (McNally et al., 2008; Dutrieux et al., 2015). TRIM14 could inhibit hepatitis C virus (HCV) replication by targeting the NS5a protein of HCV for degradation (Wang et al., 2016). TRIM52 also inhibits the Japanese encephalitis virus (JEV) replication by promoting the ubiquitin modification of NS2A protein and its degradation (Fan et al., 2016).

Latest evidence reveals that TRIM14 could also restrain IAV replication by targeting its viral nuclear proteins for degradation (Wu et al., 2019b). TRIM32 could ubiquitinate the polymerase subunit PB1 of certain IAV strains to promote its degradation and limit IAV replication (Fu et al., 2015). TRIM33 targets the viral integrase of HIV-1 to restrains its infection (Ali et al., 2019). TRIM2 interacts with the signal-regulating protein α (SIRPA), which can inhibit phagocytosis, thereby blocking New World arenaviruses (NWAs) from entering the host cell *via* endocytosis (Sarute et al., 2019). During the infection of herpesvirus, TRIM43 could induce changing of nuclear lamina by ubiquitinating and degrading centrosomal protein pericentrin, thereby restricting viral infection by inhibiting the activities of viral chromatin (Full et al., 2019).

### Viruses May Manipulate TRIM Proteins for Their Replication

**Figure 4:**
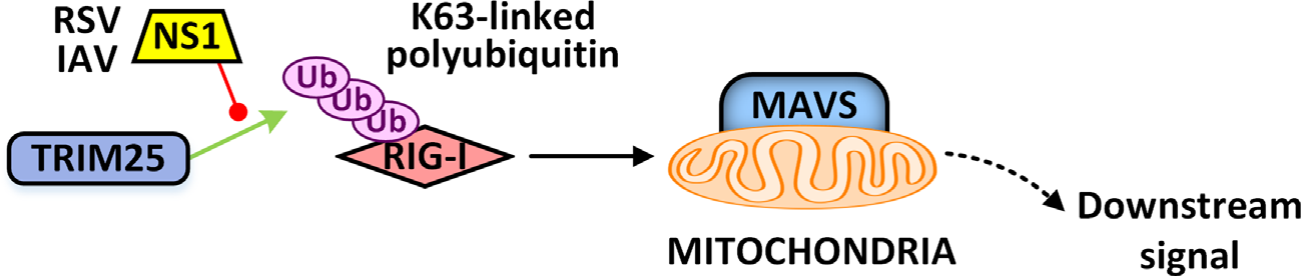
The NS1 protein of RSV or IAV prevents TRIM25-mediated activation of the RLR signaling pathway. IAV, influenza A virus; RSV, respiratory syncytial virus; NS1, nonstructural protein 1.

In the long “game” between virus and host, the virus has evolved a defense mechanism against TRIM proteins. Respiratory syncytial virus (RSV) NS1 protein could interact with the SPRY domain of TRIM25, interfering with TRIM25-mediated K63-linked polyubiquitination on the CARD domain of RIG-I to inhibit its signal transduction and preventing antiviral immune response (Ban et al., 2018). The same phenomenon and mechanism are also observed in IAV infection (**Figure 4**) (Gack et al., 2009). Nucleocapsid (N) protein of severe acute respiratory syndrome (SARS) virus (Hu et al., 2017b) and the Middle East respiratory syndrome (MERS) virus (Santiago et al., 2014) also target TRIM25 to inhibit its E3 ubiquitin ligase activity. Nucleocapsid protein of porcine reproductive and respiratory syndrome virus (PRRSV) can antagonize the antiviral activity of TRIM25 by interfering with TRIM25-mediated RIG-I ubiquitination (Chiramel et al., 2019). Through structural mimicry, the immediate early protein IE1 of human cytomegalovirus (CMV) could bind to the CCD domain of TRIM19 to prevent its auto-SUMOylation and disrupt the function of PML nucleosome. Besides, IE1 could similarly target TRIM5α and TRIM33 to inhibit their function (Francisco Puerta and Qiyi, 2014; Scherer et al., 2014; Scherer and Stamminger, 2016; Schilling et al., 2017). The ICP0 protein of HSV-1, with its own RING E3 ubiquitin ligase activity, targets TRIM27 to mediate its polyubiquitination modification and degradation (Conwell et al., 2015). The matrix protein of Nipah Virus (NiV) could target TRIM6 to reduce the synthesis of unanchored K48-linked polyubiquitin chains, thereby blocking the activation of IKKϵ and subsequent type I IFN-mediated antiviral responses (Bharaj et al., 2016).

**Figure 5:**
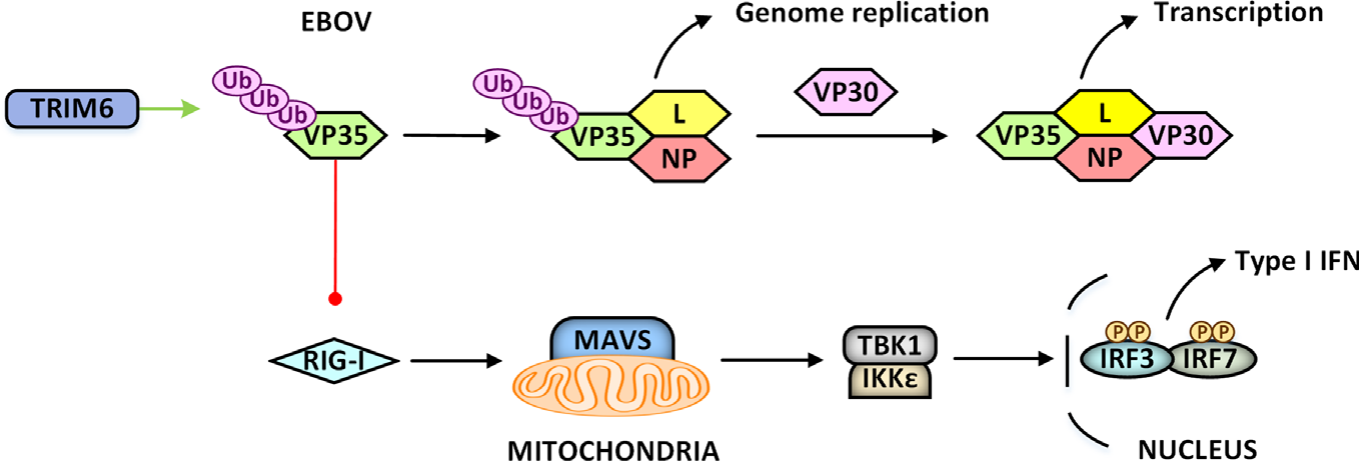
Viruses “domesticate” TRIM proteins for their replications. VP35 protein of Ebola virus (EBOV) could serve as an essential cofactor of the viral polymerase, and a potent antagonist of RIG-I mediated innate immunity. TRIM6 can be recruited to ubiquitinate VP35 protein on K309, promoting virus replication by enhancing viral polymerase activity or decreasing its ability to antagonize RIG-I mediated innate immunity.

Except for defensive, viruses can “domesticate” TRIM proteins for their replications as well. For example, TRIM21 is induced in JEV-infected human microglia cells CHME-3 to inhibit IRF3-mediated IFNβ production, thereby preventing antiviral immune response (Gunjan Dhawan et al., 2014). Epstein-Barr virus (EBV) could induce TRIM29 expression, whose E3 ubiquitin ligase activity could mediate K48-linked polyubiquitination of STING to promote its degradation and consequently inhibits activation of the cGAS-STING pathway (Xing et al., 2017). VP35 protein of Ebola virus (EBOV) could serve as an essential cofactor of the viral polymerase and a potent antagonist of RIG-I mediated innate immunity. VP35 protein can hijack TRIM6 to promote its self-polyubiquitination and then promote virus replication by enhancing viral polymerase activity or decreasing its ability to antagonize innate immunity (Bharaj et al., 2017) (**Figure 5**). UL144 protein of human cytomegalovirus interacts with TRIM23 to induce K63-linked polyubiquitination of TRAF6, activates the NF-κB signaling pathway, upregulates the expression of macrophage-derived chemokine (also known as CCL22), and finally inhibits Th1-mediated immune response (Poole et al., 2009; Emma et al., 2014). The Us11 protein of HSV-1 spatially disrupts the TRIM23-TBK1 complex, which subsequently suppresses autophagy and autophagy-mediated virus restriction (Liu et al., 2019a).

## Conclusions and Prospects

Since the first member of the TRIM protein family—*Xenopus laevis* nuclear factor 7 (XNF7)—was identified in 1991 (Reddy et al., 1991), more than 100 members (about 80 in the human genome) have been discovered in nearly 20 years, and scientists never stop exploring their structures and functions. Positioned as hubs connecting cellular signaling, autophagy, metabolism, and probably apoptosis, it is unsurprising that the TRIMs could play various roles (Mandell et al., 2020). A recent study claims that during LPS-induced inflammation in macrophages, TRIM7 can upregulate the immune response of TLR4-mediated inflammatory pathways (Lu et al., 2019). Many TRIM proteins are found as relevant cancer biomarkers, showing decreased or increased expression levels (Mandell et al., 2020). For instance, the high expression of TRIM59 in cholangiocarcinoma cells could regulate cell proliferation *via* the phosphatidylinositol-3-kinase (PI3K)/AKT/mammalian target of rapamycin (mTOR) signaling pathway (Shen et al., 2019).

Even in virus antagonism, TRIM protein is behaving multi-dimensional functions. Emerging evidence has revealed that TRIM protein could regulate virus-induced autophagy, therefore indirectly playing an antagonistic or promoting role in viral replication. Besides, several studies show that TRIM32 (Schwamborn et al., 2009), TRIM65 (Shitao et al., 2014), and TRIM71 (Chang et al., 2012) can participate in microRNA processing and RNA interfering, suggesting that TRIM proteins may potentially directly target viral-coded microRNA to affect virus replication. In short, the functions of the TRIM proteins warrant further studies.

It has been well-known that some viruses are involved in carcinogenesis, so is there any possibility that TRIM proteins participate in cancer progression by affecting virus replication? Could TRIM protein be a new molecular target for cancer therapy? Different TRIM proteins function diversely in different cell lines, and even the same TRIM protein plays opposite roles in different cell lines. What is the underlying mechanism? As the studies on TRIM proteins *in vivo* are relatively rare, and it is still unknown whether TRIM protein’s physiological roles revealed *in vivo* studies are consistent with those revealed *in vitro* studies. Elucidating the interaction and mechanism between TRIM proteins and viruses will provide new molecular targets for preventing and treating viral infectious diseases and tumors.

## Author Contributions

ZS, to write the full text, make figures, and build framework. Z-bY, to provide guidance on building framework. Z-yY and X-tS, to provide guidance on correcting grammar and rhetoric. JC, to assist to collect data. Y-tW, to assist to collect data. LW, to provide guidance on framework building and topic determination. ML, corresponding author, to modify, edit, and proofread the full text. All authors contributed to the article and approved the submitted version.

## Funding

This work has supported by the National Natural Science Foundation of China (grant numbers are 81802014 and 81801560), Scientific and Technological Research Project of Division of Science and Technology, Department of Education of Hebei Province (grant number are QN2018146 and QN2017105), and the Science and Technology Planning Project of Hebei Province (H2019206614) and The Sping Rain Program of Hebei Medical University (CYQD201812).

## Conflict of Interest

The authors declare that the research was conducted in the absence of any commercial or financial relationships that could be construed as a potential conflict of interest.
